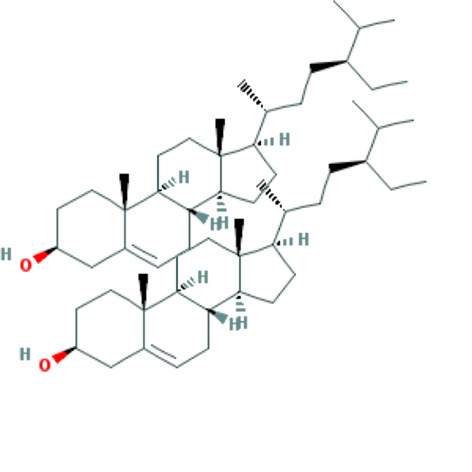# Uncovering the molecular mechanism of Mume Fructus in treatment of Sjögren’s syndrome

**DOI:** 10.1097/MD.0000000000038085

**Published:** 2024-05-10

**Authors:** Zhongli Sun, Lilin Deng, Zhoujie Xu, Kun Yang, Penglong Yu

**Affiliations:** a Chongqing Three Gorges Medical College, Chongqing, P.R. China.

**Keywords:** molecular docking, Mume Fructus, network pharmacology, Sjögren’s syndrome

## Abstract

**Background:**

Modern medicine has no cure for the xerostomia caused by the early onset of Sjögren’s syndrome. Mume Fructus is a common Chinese herbal medicine used to relieve xerostomia. However, the molecular mechanisms of the effects of Mume Fructus are unknown. In this study, network pharmacology and molecular docking were used to investigate the mechanisms of action of Mume Fructus on Sjögren’s syndrome.

**Materials and method:**

The Traditional Chinese Medicine Systems Pharmacology Database and Analysis Platform database was used to identify the active components and targets of Mume Fructus, and the UniProt database was used to identify the genes encoding these targets. SS-related targets were also identified from the GeneCards and OMIM databases. By finding the intersection of the targets of the compounds and the targets of Sjögren’s syndrome, the predicted targets of Mume Fructus in the treatment of Sjögren’s syndrome were obtained. Further investigation of the active compounds and their targets was carried out by constructing a network of “medicine-candidate compound-target-disease” using Cytoscape 3.7.2, the Protein-Protein Interaction network using the STRING database and Cytoscape 3.7.2, and key targets were identified by Gene Ontology and Kyoto Encyclopedia of Genes and Genomes enrichment analysis on R software. Finally, molecular docking was used to verify the affinity of the candidate compounds to the key targets.

**Results:**

Quercetin, beta-sitosterol, and kaempferol in Mume Fructus interact with AKT1, IL-6, IL-1B, JUN, CASP3, and MAPK8. These results suggest that Mume Fructus exerts its therapeutic effects on the peripheral gland injury of Sjögren’s syndrome and its secondary cardiovascular disease and tumorigenesis through anti-inflammatory, anti-oxidant, and anti-tumor pathways.

**Conclusion:**

With network pharmacology, this study systematically identified the main active components, targets, and specific mechanisms of the therapeutic effects of Mume Fructus on Sjögren’s syndrome, providing both a theoretical basis and research direction for further investigations on Mume Fructus.

## 1. Introduction

Sjögren’s syndrome (SS) is an autoimmune disease characterized by lymphocyte invasion of exocrine glands. It principally affects women between 40 and 60 years old.^[1]^ It is the second most common autoimmune disease endangering human health, second only to rheumatoid arthritis.^[2]^ In the early stage of SS, the functioning of the exocrine glands is damaged due to T lymphocyte infiltration and symptoms such as xerostomia, xerophthalmia, and damaged parotid glands may appear. As the disease progresses multiple systems may become involved and the discomfort caused by symptoms causes distress and often a severe psychological burden to patients. In addition, SS patients have a higher risk of developing malignant tumors and cardiovascular disease.^[3–5]^

Modern medicine has no cure for the xerostomia symptoms caused by the early onset of SS. Topical salivary replacement therapies such as gels, sprays, and buffers have been regarded as the first choice for relieving xerostomia, but there is no evidence that they can effectively relieve the discomfort of patients.^[6]^

Immunosuppressants are not recommended for the treatment of early SS xerostomia due to the risk of immune disorders, tumor, liver, and kidney damage.^[7]^ Additional symptoms accompanying xerostomia, including appetite disorders and dental caries, can bring serious inconvenience to the patients’ lives. Drinking large amounts of water to ease the dryness of the mouth may also increase the burden on the kidneys.

In recent years, studies have shown that traditional Chinese medicine therapy can effectively reduce inflammatory indicators such as IL-6, IL-10, erythrocyte sedimentation rate, and C-reactive protein in the peripheral blood of SS patients and can prevent further aggravation of the disease while relieving local symptoms.^[8,9]^ In addition, Chinese herbal medicine can also upregulate the expression of aquaporin (AQP)-5 through a variety of ways (including immune regulation, suppression of inflammation, and inhibition of the destruction of surrounding glands), effectively increase the secretion of surrounding glands, and significantly improve the symptoms of xerostomia and xerophthalmia.^[10–12]^ Therefore, many SS patients in China choose to use Chinese herbal medicine for treatment.

Mume Fructus (unripe fruit of Prunus mume (Siebold) Siebold & Zucc.) is a common Chinese herbal medicine used to relieve xerostomia. It is recorded in the medical book *Gleaning Herb* of the Tang Dynasty (618-97) that Mume Fructus was used to treat xerostomia. In clinical treatment, Mume Fructus preparations are often used to relieve xerostomia caused by diabetes,^[13,14]^ radiotherapy, and adverse reactions to atropine. Literature analysis shows the importance of Mume Fructus in the treatment of SS and it is reported to relieve the symptoms of xerostomia, xerophthalmia, fever, and body pain caused by SS.^[15–17]^

**Figure 1.**
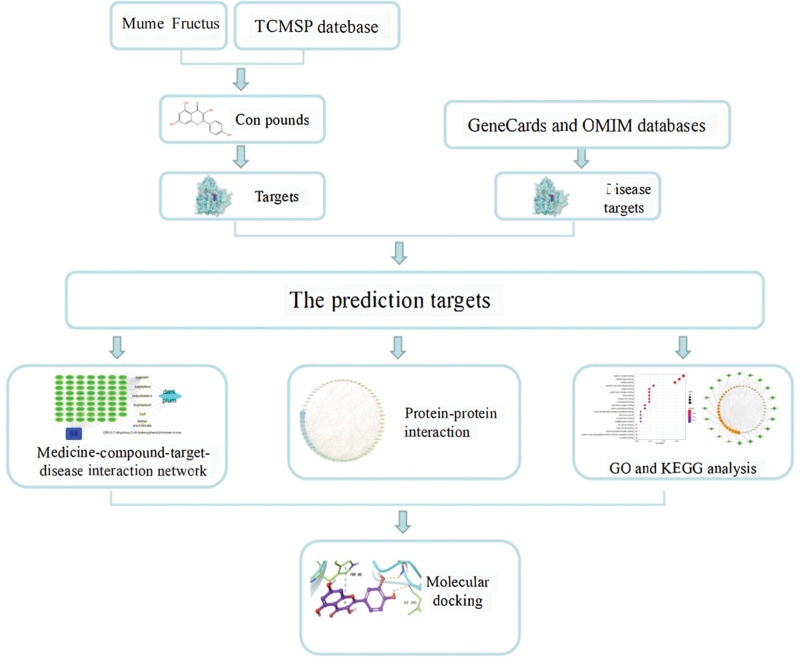
Work-flow chart.

As an autoimmune disease, SS has a complex pathogenesis and its symptoms can vary when affected by various factors. As a plant-derived medicine, Mume Fructus consists of a variety of components and could exert its therapeutic effect on SS symptoms in different ways and through multiple targets.^[18,19]^ There are few studies on the mechanism of action of Mume Fructus therapy for SS, and the active components are still unclear. Therefore, this study adopted a multi-component, multi-target, and multi-pathway research method of network pharmacology to study the effective components, therapeutic targets, and mechanisms of the treatment of SS with Mume Fructus (Fig. 1). The aim is to elucidate the mechanisms involved in the treatment of SS with Mume Fructus and to provide a theoretical basis and research directions for the treatment of SS with Mume Fructus.

## 2. Materials and methods

### 2.1. The effective components and targets of Mume Fructus as identified from the TCMSP database

The components of Mume Fructus were obtained by using “Mume Fructus” as the keyword in searching the Traditional Chinese Medicine Systems Pharmacology Database and Analysis Platform (TCMSP) (https://www.tcmsp-e.com/#/home). Taking oral bioavailability (OB) ≥ 30% and drug-likeness (DL) ≥ 0.18 as the screening conditions, the effective components of Mume Fructus were selected as candidate compounds.^[20,21]^

The targets of the candidate compounds were also identified from the TCMSP database, and the human genes encoding the target molecules were identified from the UniProt database (http://www.uniprot.org/); the target molecules are henceforth referred to by these genetic names.

### 2.2. Identifying the SS-related target genes in the GeneCards and OMIM databases

In the GeneCards database (https://www.genecards.org/) and OMIM database (https://omim.org/), the SS-related targets were identified by using “Sjögren’s syndrome” as keywords and the results compared.

### 2.3. Establishing the “medicine-candidate compound-target-disease” network by using Cytoscape 3.7.2 and analyzing the results

The molecular targets of the candidate compounds of Mume Fructus were pooled with the SS-related targets, and the results were used as the prediction targets of the therapeutic effects of Mume Fructus on SS. Cytoscape 3.7.2 (https://cytoscape.org/) software was used to establish a network of “medicine-candidate compound-target-disease,” and the results were analyzed. In the network, the nodes represented Mume Fructus, candidate compound, potential target, and disease. The edges represented the association between Mume Fructus, candidate compounds, potential targets, and disease. By analyzing the network, the main components to cure SS were identified.

### 2.4. Using the STRING database and Cytoscape 3.7.2 to build the PPI network and analyzing the results

The STRING database was used to analyze the prediction targets of Mume Fructus on SS. The species in question was designated *Homo sapiens*, with a minimum interaction score of 0.4. The remaining parameters adopted the default values to establish the Protein-Protein Interaction (PPI) network. Cytoscape 3.7.2 software was used to rank and analyze the PPI network, and “high degree target” was selected as the key target.

### 2.5. GO enrichment analysis and KEGG enrichment analysis

R software (R.3.6.1 for Windows) was used for the GO and KEGG enrichment analyses of the prediction targets of Mume Fructus on SS. The key gene construction network of KEGG enrichment analysis was sequenced by using Cytoscape 3.7.2 software to screen out the key targets in the signal pathway.

### 2.6. Molecular docking

ChemBioDraw Ultra 17.0 was used to draw the two-dimensional structure of the main compounds of Mume Fructus, which was then transformed into a three-dimensional structure by using ChemBio3D Ultra 17.0, and was optimized with the MMFF94 force field.

The PPI network was incorporated with the top 10 genes in degree in KEGG, and the overlapping genes were used as the targets for molecular docking. The three-dimensional structure of the pre-targets was downloaded from the RCSB Protein Data Bank (www.rcsb.org).The corresponding ligands were found in RCSB as positive reference.

The three-dimensional structures of the pre targets and compounds were transformed into PDBQT format using AutoDockTools 1.5.6.^[22]^ AutoDock Vina 1.1.2^[23]^ was used for molecular docking. To increase the accuracy of the calculation, the “exhausitiveness” parameter was set to 20, with the other parameters adopting the default values. Finally, the highest-scoring conformation was selected and analyzed with Maestro 11.9 (Schrödinger).

## 3. Results

### 3.1. Candidate compounds and targets of Mume Fructus

Forty Mume Fructus compounds were identified in the TCMSP database. Of these, 8 met the screening criteria of OB ≥ 30% and DL ≥ 0.18. The basic information of these candidate compounds is listed in Table 1 and Figures S1–S8, Supplemental Digital Content, http://links.lww.com/MD/M407, http://links.lww.com/MD/M408, http://links.lww.com/MD/M409, http://links.lww.com/MD/M410, http://links.lww.com/MD/M411, http://links.lww.com/MD/M412, http://links.lww.com/MD/M413, http://links.lww.com/MD/M414 in the order of descending OB values.

**Table 1:** Information on candidate compounds.

MOL ID	Molecule name	Structure	OB (%)	DL
MOL008601	methyl arachidonate	See supplemental figure 1	46.90	0.23
MOL000098	quercetin	See supplemental figure 2	46.43	0.28
MOL000449	stigmasterol	See supplemental figure 3	43.83	0.76
MOL001040	(2R)-5,7-dihydroxy-2-(4-hydroxyphenyl)chroman-4-one	See supplemental figure 4	42.36	0.21
MOL000422	kaempferol	See supplemental figure 5	41.88	0.24
MOL000953	CLR	See supplemental figure 6	37.87	0.68
MOL005043	campest-5-en-3beta-ol	See supplemental figure 7	37.58	0.71
MOL000358	beta-sitosterol	See supplemental figure 8	36.91	0.75

One hundred sixty-four targets of these eight candidate compounds were obtained from the TCMSP database and their human gene codes identified in the UniProt database.

### 3.2. Identifying SS targets in the GeneCards and OMIM databases

Using “Sjögren’s syndrome” as the keywords, 784 and 1336 targets were identified in the *GeneCards* and OMIM databases, respectively, and 1395 targets related to SS were obtained after removal of duplicates.

### 3.3. Analysis of the results of “medicine-compound-target-disease” network

Pooling the 164 Mume Fructus targets from the TCMSP database with the 1395 SS targets resulted in an overlap of 62 potential targets, relating to the 7 candidate compounds.

**Figure 2.**
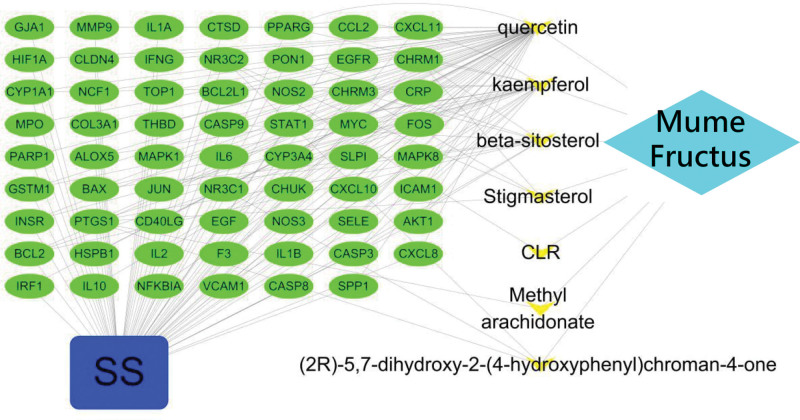
Medicine-compound-target-disease interaction network.

The “medicine-compound-target-disease” network was established by using the Cytoscape 3.7.2 software. There were 71 nodes in the network (including 7 nodes of the compounds, 62 nodes of the target genes, 1 node of the disease, and 1 node of the medicine) and 165 edges (Fig. 2). The more edges the node connected, the higher the degree value. It can be seen that the key compounds in the network were quercetin (degree = 57), kaempferol (degree = 22), and beta-sitosterol (degree = 11).

### 3.4. PPI network result analysis

In the STRING database, the 62 predicted targets were imported to establish the PPI network. In the network, protein-protein interactions of the 62 targets were identified with 773 lines representing interactions.

**Figure 3.**
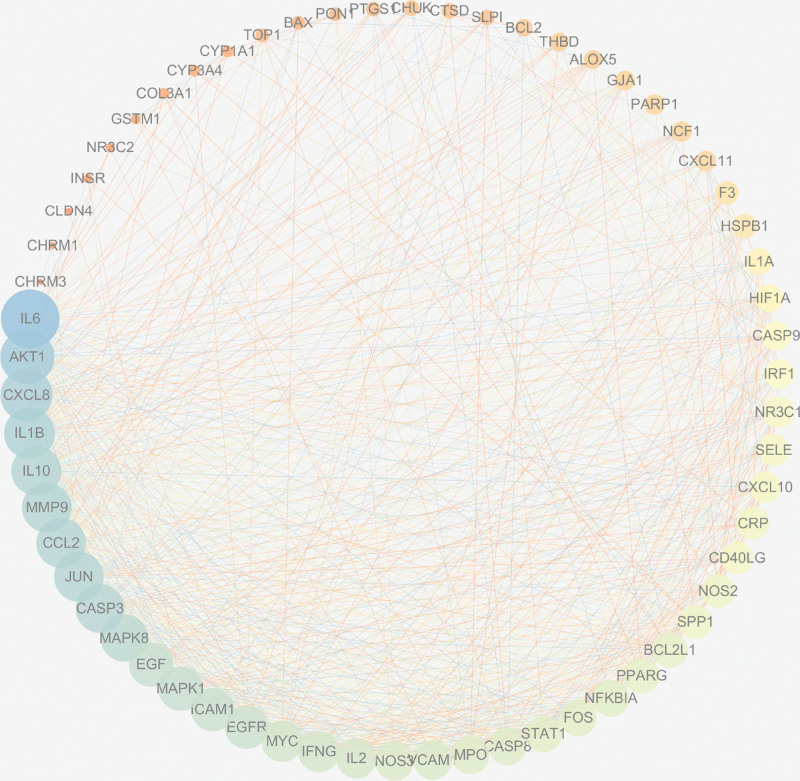
PPI network. PPI = protein-protein interaction.

Cytoscape 3.7.2 software was used to rank the PPI network results in order of degree (Fig. 3). The top 10 proteins in the PPI network were found to be interleukin (IL)6 (degree = 52), protein kinase B (AKT) 1 (degree = 48), C-X-C motif chemokine ligand 8 (degree = 46), interleukin-1 beta (IL-1B) (degree = 45), chemokine (C-C motif) ligand 2 (degree = 44), interleukin (IL)10 (degree = 44), Jun proto-oncogene, AP-1 transcription factor subunit (JUN) (degree = 44), matrix metallopeptidase 9 (degree = 44), caspase (CASP) 3 (degree = 43), and mitogen-activated protein kinase (MAPK) 8 (degree = 42).

### 3.5. Analysis of enrichment analysis results

**Figure 4.**
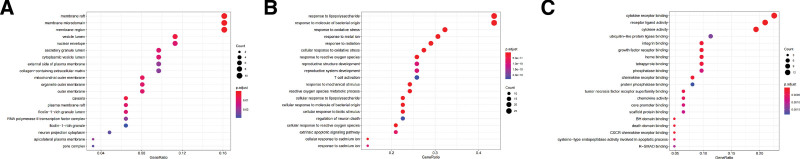
GO enrichment analysis results. GO = gene ontology.

GO enrichment analysis showed that the 62 predicted target genes participated in 1532 biological processes through 86 molecular functions (*P* < .05, *q* < 0.05). In the enrichment analysis results, according to the adjust *P* value and target count, we can see that these genes are mainly distributed in the membrane raft, the membrane microdomain, and the membrane region (Fig. 4A), they can respond to lipopolysaccharide, a molecule of biological origin associated with oxidative stress (Fig. 4B), and the main biological processes involved include cytokine receptor binding, receptor ligand activity, and cytokine activity (Fig. 4C).The size of the dots in the graph represents the number of predicted target genes enriched in this term, and the color represents the value of adjust *P*. A shows the results of cellular component (CC), B shows the results of biological process, and C shows the results of molecular function (MF).

**Figure 5.**
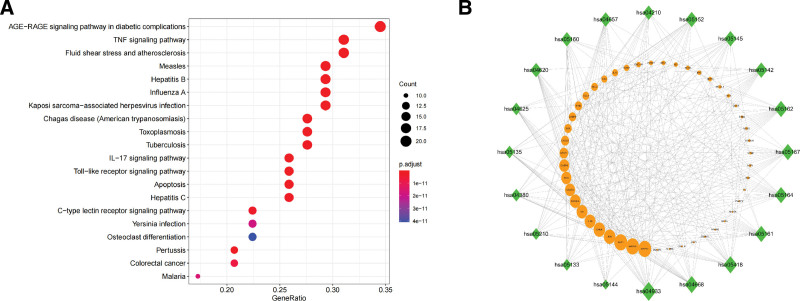
Analysis results of KEGG enrichment. KEGG = Kyoto Encyclopedia of Genes and Genomes.

The results of the KEGG enrichment analysis showed that 144 pathways were affected by the 62 predicted target genes (*P* < .05, *q* < 0.05). According to the value of adjust P and target count, the most critical paths include the GE − RAGE signaling pathway in diabetic complications, the TNF signaling pathway, and pathways involved in fluid shear stress and atherosclerosis (Fig. 5A). The “gene- pathway” network was constructed by using the software of Cytoscape 3.7.2. According to the ranking results of degree, MAPK1 (degree = 17), MAPK8 (degree = 17), AKT1 (degree = 17), JUN (degree = 15), CHUK (degree = 15), IL-1B (degree = 14), IL-6 (degree = 14), NFKB1A (degree = 14), CASP3 (degree = 13), and FOS (degree = 13) play important roles in multiple pathways (Fig. 5B).A shows the predicted target gene involved pathways. The size of the dots represents the number of target genes enriched in the pathway, and the color represents the adjust *P* value. B shows the predicted gene affected pathways. The ellipse represents the predicted target gene, and the diamond represents the signal pathway. The higher the degree value, the larger the graph.

### 3.6. Analysis of molecular docking results

The results of the “medicine-compound-target-disease” interaction network showed that the compounds of high value in the treatment of SS were quercetin, kaempferol, and beta-sitosterol.

The overlapped genes of the top 10 genes in the PPI network and those in the KEGG enrichment were AKT1, IL-6, IL-1B, JUN, CASP3, and MAPK8.

**Figure 6.**
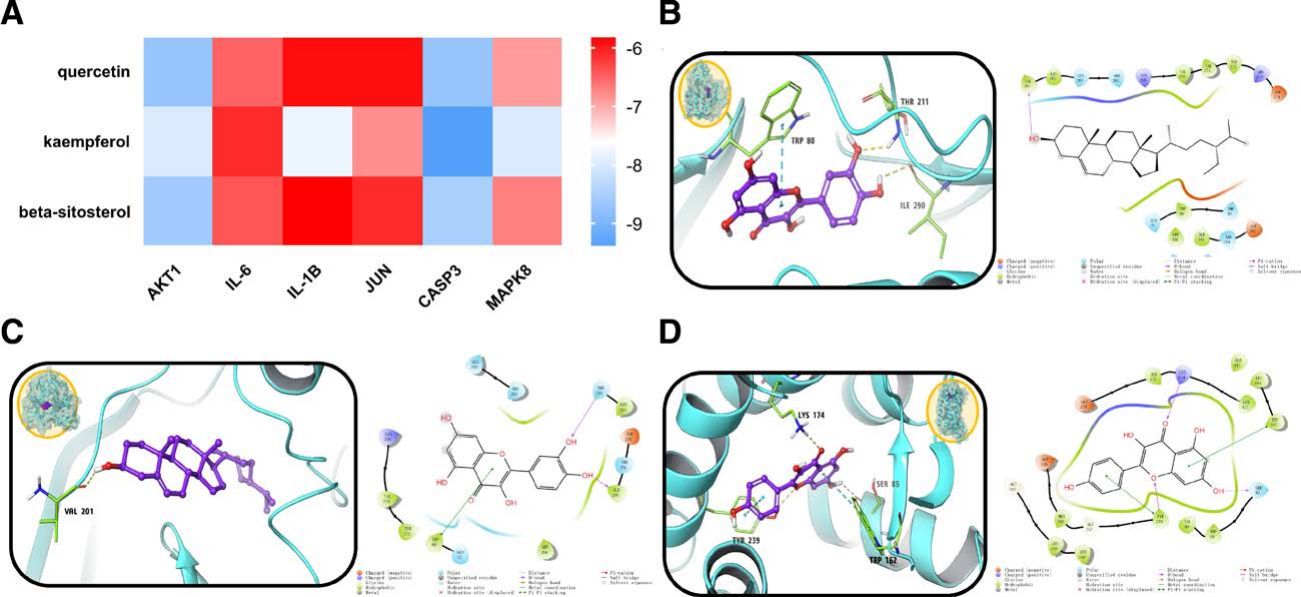
Interactions between quercetin, kaempferol, and beta-sitosterol with target proteins.

After obtaining the three-dimensional structure of the above compounds and genes, molecular docking was carried out. We calculated the binding degrees of AKT1, IL-6, IL-1B, CASP3, and MAPK 8 (JUN could not find the corresponding ligand in the database)to their ligands (Table 2). In order to elucidate the interaction mechanisms of quercetin, kaempferol, and beta-sitosterol with AKT1, IL-6, IL-1B, JUN, CASP3, and MAPK 8 at the molecular level, we docked the compounds quercetin, kaempferol, and beta-sitosterol to the active pockets of the protein targets AKT1, IL-6, IL-1B, JUN, CASP3, and MAPK8, and compiled an affinity heatmap (Fig. 6A). The results showed that the three combinations with highest affinity were CASP3-kaempferol, AKT1-beta-sitosterol, and AKT1-quercetin. The mapping results of these three pairs showed that the small molecules are closely bound to the surface of the proteins, indicating that there is a good match between them.

**Table 2:** Binding degree of corresponding ligands.

Target	PDBID	Ligand	Formula	Degree
AKT1	4KEL	(2S)-2-(4-chlorophenyl)-1-{4-[(5R,7R)-7-hydroxy-5-methyl-6,7-dihydro-5H-cyclopenta[d]pyrimidin-4-yl]piperazin-1-yl}-3-(propan-2-ylamino)propan-1-one	C_24_H_32_ClN_5_O_2_	−8.1
IL1B	5R86	~{N}-(4-hydroxyphenyl)-2-methoxy-ethanamide	C_9_H_11_NO_3_	−5.5
IL6	1ALU	L(+)-TARTARIC ACID	C_4_H_6_O_6_	−4.7
CASP3	1RHJ	3-(2-{5-TERT-BUTYL-3-[(4-METHYL-FURAZAN-3-YLMETHYL)-AMINO]-2-OXO-2H-PYRAZIN-1-YL}-BUTYRYLAMINO)-5-(HEXYL-METHYL-AMINO)-4-OXO-PENTANOIC ACID ANION	C_28_H_44_N_7_O_6_	−4.4
MAPK8	2XRW	PHOSPHOAMINOPHOSPHONIC ACID-ADENYLATE ESTER	C_10_H_17_N_6_O_12_P_3_	−7.0

Quercetin binds to the active site of AKT1, and forms hydrogen bonds with two of the active site residues, Thr211 and Ile290, while forming π-π interactions with Trp80 (Fig. 6B). Beta-sitosterol also binds to the active site of AKT1, with the hydroxyl group at one end of the compound interacting with Val201 to form a hydrogen bond, while the bulky region at the other end is located in the active cavity where it forms hydrophobic interaction with the protein (Fig. 6C). Kaempferol binds to the active site of CASP3, forming hydrogen bonds with Lys174, Ser85, and Tyr239, and π-π interactions with Tyr239 and Trp162 (Fig. 6D). Hydrogen bond interactions are critical in the interactions between proteins and compounds, promoting the formation of stable complexes, and provide evidence for the therapeutic effects of quercetin, beta-sitosterol, and kaempferol on SS.

A: Heatmap of binding interactions. The abscissa represents the target, the ordinate represents the compound, and the color represents the binding degree. Higher absolute values indicate better binding.

B: Action mode of Quercetin and AKT1.

C: Action mode of Beta-sitosterol and AKT1.

D: Action mode of Kaempferol and CASP3

## 4. Discussion

In this study, the active components, targets, and mechanisms of Mume Fructus in treating SS were investigated by means of network pharmacology and molecular docking.

Firstly, the “medicine-compound-target-disease” interaction network was constructed to analyze the effective components and c*o*rresponding targets of Mume Fructus in the treatment of SS. The results showed that quercetin, kaempferol, and beta-sitosterol were the key active compounds. As an autoimmune disease, the exocrine gland injury of SS is closely related to lymphocyte infiltration.Existing studies have shown that T cell subsets such as Th1, Th2, Th17, and related cytokines secreted by B cells, such as TNF-α, IL-6, IL-17, IL-1, may participate in the damage process of peripheral secretory glands of SS.^[24]^These cytokines can also affect AQP5 through NF-κB, MAPK and other signaling pathways, thus aggravating xerostomia and xerophthalmia.^[25]^Quercetin has been shown to reduce the production of inflammatory factors through the NF-κB and MAPK pathways.^[26,27]^ In addition, quercetin has a significant immunoregulatory effect, and it can inhibit the abnormal proliferation and activation of T lymphocytes and improve the chronic inflammation caused by the infiltration of T lymphocytes in the early stage of SS.^[28,29]^ Kaempferol can regulate the Th1/Th2 cell subpopulation to achieve the goal of immune regulation.^[30]^ Beta-sitosterol reduces the expression of inflammatory factors such as IL-6 and IL-1, regulates cell numbers in the Th1 cell subpopulation, and regulates the overall state of immunity.^[31]^Therefore, quercetin, kaempferol, and beta-sitosterol can reduce the expression of inflammatory factors, reduce the damage of exocrine glands, and alleviate the early symptoms of SS.

SS patients have a higher risk of malignant tumor and cardiovascular disease in the later stage of the disease. Quercetin, kaempferol, and beta-sitosterol have additional effects, specifically, as anti-oxidants and anti-tumor agents,^[32–34]^ reducing the risks of both tumors and cardiovascular disease in patients with advanced SS. Mume Fructus can, therefore, not only reduce the peripheral glandular damage of early SS by modulating inflammation, but also has certain preventive effects on the secondary cardiovascular and cerebrovascular complications of SS.^[35–37]^

The results of the PPI, GO enrichment analysis, and KEGG enrichment analysis show that Mume Fructus is likely to have therapeutic effects on SS through multiple targets and mechanisms. According to the PPI network analysis and the KEGG results, the most critical targets of Mume Fructus in the treatment of SS are AKT1, IL-6, IL-1B, JUN, CASP3, and MAPK8. These genes play an important role in cytokine activity, inflammatory response, cell proliferation, apoptosis, and tumorigenesis. Clinical research has confirmed that IL-6 participates in the pathogenesis of SS and is related to the disease activity of it.^[38,39]^ IL-6 is also considered to be an important mediator of coronary heart disease, and its expression is closely related to the occurrence of cardiovascular disease. In addition, IL-6 can aggravate tumor invasion through its role in the STAT3-Twist signaling pathway. It has been suggested that IL-6 may play an important role in the disease activity of SS, as well as the secondary tumorigenesis and cardiovascular disease. IL-1B participates in the destruction of peripheral glands by mediating inflammation and may further participate in the pathogenesis of SS by affecting the differentiation of Th17 cells. In addition, IL-1B is considered to be the central link in acute coronary syndrome, and it may be closely related to the occurrence of cardiovascular disease in the later stages of SS. JUN and MAPK8 can affect the symptoms of xerostomia and xerophthalmia in SS patients by regulating the expression of AQP5. There are few studies on the relation between AKT1, CASP3, and SS. As classical genes, AKT1 and CASP3 may be related to the later stages of SS.^[40]^

The results of the molecular docking showed that quercetin, beta-sitosterol, and kaempferol had good affinities with AKT1, IL-6, IL-1B, JUN, CASP3, and MAPK8, resulting in stable complexes on binding. The hydrogen bond and π-π interactions between complexes may be the key to the effect of compounds on proteins. This explains the mechanism of the therapeutic effects of the main active components of Mume Fructus, namely, quercetin, beta-sitosterol, and kaempferol, on SS.

Many cytokines and pathways have been found to be closely related to the pathogenesis of SS. For example, as a representative cytokine, IL-17 participates in the damage of the peripheral glands and is highly expressed in the peripheral blood of SS patients In addition to participating in peripheral gland inflammation, TNF-α also affects the expression of AQP5 in peripheral secreting glands through the NF-κB pathway and has been shown to influence the xerostomia and xerophthalmia symptoms in SS patients.^[41]^

As an autoimmune disease, the specific pathogenesis of SS has not been clarified. At present, there are many pathways that cannot be proved to have direct relationships with SS. For example, although there is no direct evidence that the AGE-RANGE signaling pathway in diabetic complications is related to SS, it can activate NF-κB and regulate many inflammatory factors. Diabetic patients may be affected by NF-κB pathway to develop xerostomia similar to that seen in SS patients., suggesting that this may be the role of the AGE-RAGE signaling pathway in the pathogenesis of SS. Considering Kaposi’s sarcoma-associated herpesvirus (HHV-8), there is no obvious evidence that HHV-8 infection is necessarily related to SS.^[42]^ However, HHV-8 can directly encode a kind of interleukin-6 (vll-6) and induce the expression of IL-6 in the human body, which may account for the high expression of IL-6 in some HHV-8-infected patient.^[43]^ This suggests that HHV-8 infection may participate in SS process through its effect on IL-6. Further studies on on signaling pathway may provide new directions for investigation of the pathogenesis of SS.

In this study, it has been confirmed that Mume Fructus can have a variety of therapeutic effects on SS from interactions between its active components with multiple targets. However, the study did not extend to the effects of dosage forms and doses on efficacy. In addition, the possibility that the other components of Mume Fructus may have therapeutic effects on SS remains to be verified by further experiments.

This indicates that Mume Fructus can be used as a drug to relieve the symptoms of dry mouth caused by SS, and may also prevent cardiovascular diseases secondary to SS, and have certain anti-tumor effects. If it is further developed as a health food or drug for SS, it may have a huge market potential.

## 5. Conclusion

With the help of network pharmacology, this paper systematically elaborated the main active components, targets, and related mechanisms of SS treatment with Mume Fructus. The research method of network pharmacology combined with molecular docking can be used as an effective model to study the mechanism of Chinese herbal medicine against disease. The results showed that Mume Fructus could produce therapeutic effects on the peripheral gland injury of SS and its secondary cardiovascular diseases and tumors by means of anti-inflammatory, antioxidant, and anti-tumor functions.

These results provide theoretical basis and further research direction for the treatment of SS with Mume Fructus.

## Author contributions

**Data curation:** Penglong Yu

**Formal analysis:** Lilin Deng

**Methodology:** Kun Yang

**Supervision:** Zhoujie Xu

**Writing – original draft:** Zhongli Sun

**Writing – review & editing:** Kun Yang

## Supplementary Material

**Figure:**
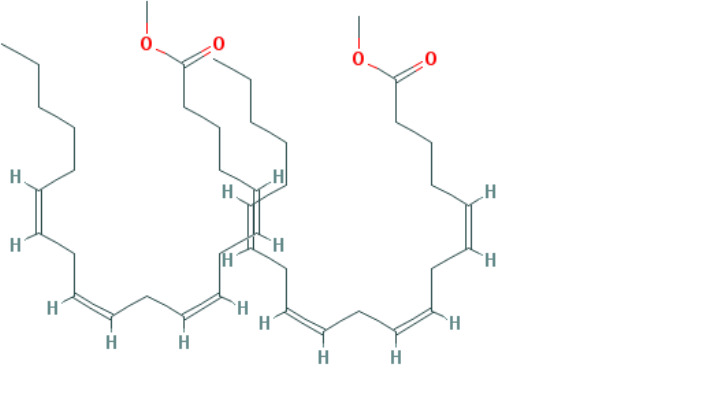


**Figure:**
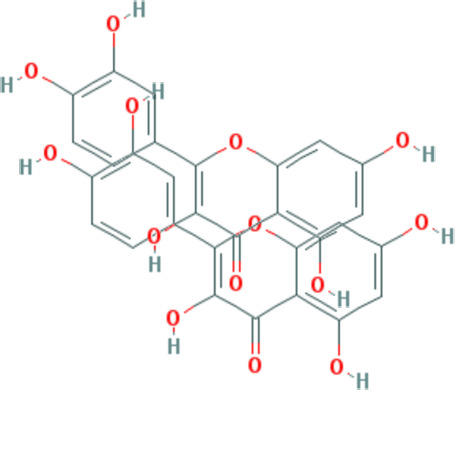


**Figure:**
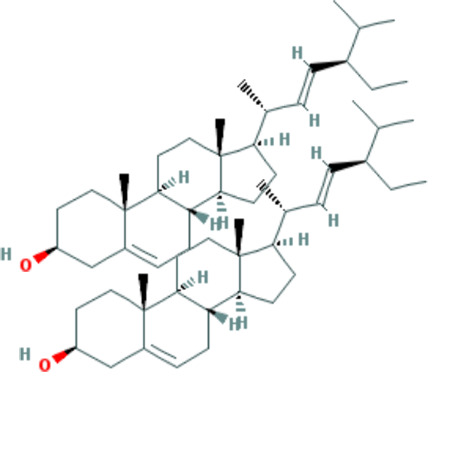


**Figure:**
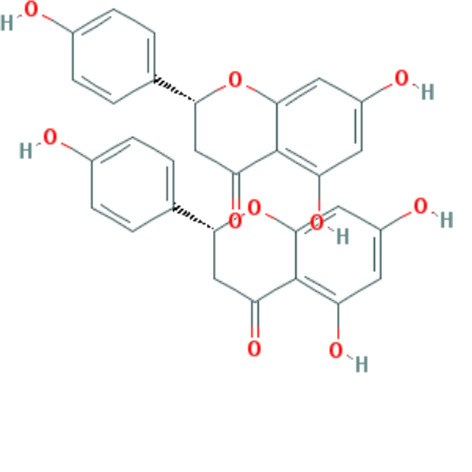


**Figure:**
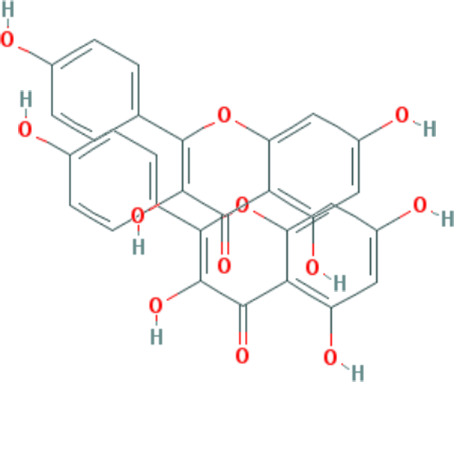


**Figure:**
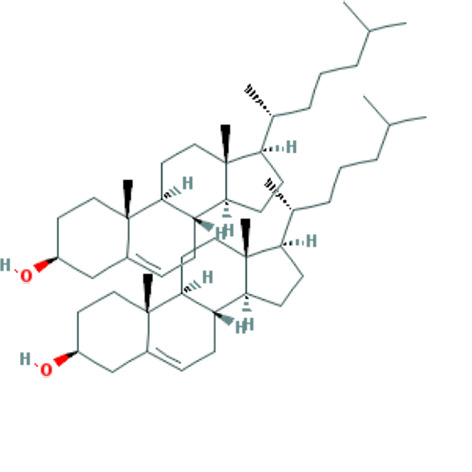


**Figure:**
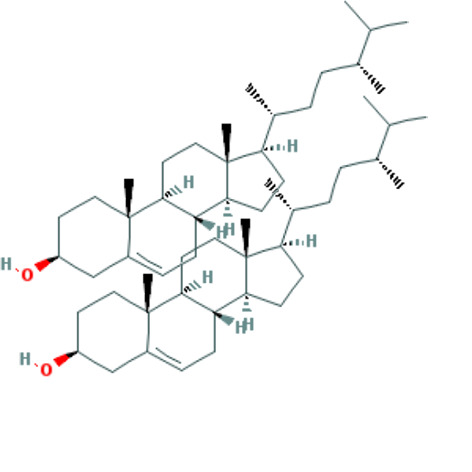


**Figure:**